# Cabozantinib Plus Nivolumab in Adult Patients with Advanced or Metastatic Renal Cell Carcinoma: A Retrospective, Non-Interventional Study in a Real-World Cohort/GUARDIANS Project

**DOI:** 10.3390/cancers16172998

**Published:** 2024-08-28

**Authors:** Thomas Hilser, Christopher Darr, Günter Niegisch, Marco Julius Schnabel, Susan Foller, Lorine Häuser, Stefanie Zschäbitz, Jonas Lewerich, Philipp Ivanyi, Katrin Schlack, Pia Paffenholz, Eveline Daetwyler, Dora Niedersüß-Beke, Viktor Grünwald

**Affiliations:** 1 West German Tumor Center Essen, Department of Internal Medicine, University Hospital Essen, 45147 Essen, Germany; 2 Department of Urology, University Hospital Essen, 45147 Essen, Germany; 3 Department of Urology, Medical Faculty, University Hospital Düsseldorf, Heinrich-Heine-University Düsseldorf, 40225 Düsseldorf, Germany; 4 Centre for Integrated Oncology (CIO) Düsseldorf, CIO Aachen-Bonn-Cologne-Düsseldorf, 50937 Köln, Germany; 5 Department of Urology, University Regensburg, Caritas-Hospital St. Josef, 93053 Regensburg, Germany; 6 Department of Urology, University Hospital Jena, 07747 Jena, Germany; 7 Department of Urology and Neuro-Urology, Marien Hospital Herne, Ruhr-University Bochum, 44625 Herne, Germany; 8 National Center for Tumor Diseases, Department of Medical Oncology, University Hospital Heidelberg, 69120 Heidelberg, Germany; 9 Department of Urology, Klinikum Rechts der Isar, Technical University Munich, 81675 Munich, Germany; 10 Department of Hemostaesiology, Oncology and Stem Cell Transplantation, Medical University Hannover, 30625 Hannover, Germany; 11 Claudia von Schelling Center, Comprehensive Cancer Center Hannover, 30625 Hannover, Germany; 12 Department of Urology, University Hospital Muenster, 48149 Muenster, Germany; 13 Department of Urology, Uro-Oncology, Robot Assisted and Reconstructive Urologic Surgery, Faculty of Medicine, University of Cologne, University Hospital Cologne, 50937 Cologne, Germany; 14 Division of Medical Oncology and Hematology, Cantonal Hospital St. Gallen, 9000 St. Gallen, Switzerland; 15 1st Department of Medical Oncology and Haematology, Klinik Ottakring, 1160 Vienna, Austria

**Keywords:** metastatic renal cell carcinoma, real-world data, immunotherapy, cabozantinib

## Abstract

**Simple Summary:**

ICI-based combinations have led to a significant change in mRCC medical treatment. However, data from real-world (RW) cohorts are rare. In this multicenter study, we evaluated the safety and effectiveness of cabozantinib plus nivolumab in real-world cohorts from centers in Germany, Switzerland, and Austria. The median PFS in the overall cohort was 18.6 months. We also analyzed subgroups in relation to the IMDC, histology, and with regard to the presence of bone metastasis. In summary, our real-word data support the promising efficacy data of the pivotal trials, particularly in patients with non-ccRCC and those without bone metastases at the start of the treatment.

**Abstract:**

Introduction: Combinations of immune-checkpoint inhibitors (ICIs) are the standard of care (SOC) for treatment-naive metastatic renal cell carcinoma (mRCC) patients. In this multicenter study, we evaluated the RW safety and efficacy of cabozantinib plus nivolumab in mRCC patients. Methods: Data were retrospectively collected from twelve cancer centers in Germany, Switzerland, and Austria. Patients with advanced or mRCC were eligible. The investigator-based objective response rate (ORR) and progression free survival (PFS) were calculated from the start of the treatment to progression or death. Descriptive statistics and Kaplan–Meier (KM) plots were utilized where appropriate. Results: In total, 96 eligible patients (66.6% male) with a median age of 66.0 years were included. The most common histology was clear-cell RCC (ccRCC) in 63.4% (*n* = 61). A prior nephrectomy was performed in 60.4% (*n* = 58). ECOG 0-1 was 68.8% (*n* = 66). A partial response was documented in 43.8% of patients (*n* = 42), a stable disease in 32.3% (*n* = 31), and a progressive disease in 8.3% (*n* = 8) as the best overall response. Response data were not evaluable in 13.5% (*n* = 13). The median follow-up time was 12.7 months (95% CI, 10.0–15.3). The PFS rate at 6 months was 89.8% in the overall population (86.8% for ccRCC; 90.0% for non-ccRCC). Adverse events (AEs) were reported in 82.3% (*n* = 79) for all grades and 41.7% (*n* = 40) for grades 3–5. Elevated liver enzymes (34.4%), diarrhea (31.3%), and hand–foot syndrome (29.2%) were the three most frequent AEs of any grade and causality. Discussion/Conclusions: In this real-world cohort of mRCC patients, the application of cabozantinib plus nivolumab was shown to be safe and feasible. Our data support the use of cabozantinib plus nivolumab as a first-line standard therapy in mRCC patients. Major limitations were the retrospective data capture and short follow-up time of our study.

## 1. Introduction

Renal cell carcinoma (RCC) accounts for 2% of tumor diseases worldwide, and its incidence has been increasing over the past decades [1,2]. Histologically, clear-cell renal cell carcinoma (ccRCC) is the dominant subtype, accounting for 75–80% of renal cancers [3]. Antisystemic cancer treatment is the main approach in patients with advanced, metastatic, or recurrent RCC who are not candidates for local therapies. ccRCC in particular is resistant to conventional chemotherapy [4]. RCC is characterized by increased angiogenesis, caused by loss of the VHL gene [5]. So, tyrosine kinase inhibitors (TKIs), targeting the vascular endothelial growth factor receptor (VEGFR), and ICIs have become pivotal in the systemic treatment of advanced or metastatic renal cell carcinoma (mRCC).

Cabozantinib is a small-molecule inhibitor of tyrosine kinases, and nivolumab is a programmed death 1 (PD-1) immune-checkpoint inhibitor antibody [6,7,8,9]. Cabozantinib inhibits tyrosine kinasis involved in tumor cell proliferation, neovascularization, and immune cell regulation. Moreover, cabozantinib has immunomodulatory properties, which may enhance the response to immune-checkpoint inhibition [10,11,12,13].

Recently, ICI-based combinations have been developed, leading to a significant change in mRCC medical treatment [14,15,16]. Currently, there are five approved first-line combinations, including the combination of cabozantinib plus nivolumab.

The combination of ICIs and TKIs is established in the first-line treatment of mRCC and includes the possibility of combining pembrolizumab with lenvatinib [17], pembrolizumab with axitinib [18], and avelumab with axitinib [19]. The current long-term data of the respective approval study confirm the clinical benefit [18,20,21]. Furthermore, the CheckMate 9ER trial investigated the combination of cabozantinib plus nivolumab as a first-line therapy in mRCC patients. The progression free survival (PFS) of this combination was significantly longer compared to sunitinib at 16.6 months versus 8.3 months [22]. With a median follow-up of 55 months, the most recent analysis presented mature survival data. The median overall survival was 46.5 versus 36.0 months in favor of the ICI combination [HR 0.77 (95% CI 0.63–0.95)] [23]. This effect was also shown in patients with papillary, unclassified, or translocation-associated RCC. In the phase II trial of cabozantinib plus nivolumab, the median PFS of patients with non-clear-cell RCC was increased to 12.5 months (95% CI 6.3–16.4) [24]. These results of the aforementioned studies continue to support cabozantinib plus nivolumab as a standard of care for previously untreated mRCC.

However, the wide range of medical treatment options allows for individualized therapy selection to identify the most appropriate treatment option for a patient. The absence of a direct comparison between contemporary ICI combinations renders the interpretation of clinical data important. The complete response (CR) and deep response after systemic treatment are associated with the best long-term results in patients with mRCC. With the current follow-up of 55 months, the CR rate for cabozantinib plus nivolumab was 13.6% (sunitinib 4.6%) [23]. ICI–TKI combinations offer a broad clinical activity with a high response rate (55–71%). The potential of these combinations becomes more evident when the low primary progression rate (5–10%) is taken into account. This broad activity has led to the development of the most effective treatments of mRCC with a median PFS in the range of 15–22 months. These strengths of the ICI–TKI combinations are of particular interest in treating symptomatic patients. Combinations with 3rd-generation TKIs (cabozantinib) have a low progression rate (6.5%) and therefore offer advantages for particularly vulnerable patients. Nevertheless, ICI–TKI combinations also have the highest rates of grade ≥ 3 AEs. However, there are differences between the ICI–TKI combinations in the incidence rate of specific AEs.

Data from real-world cohorts are rare. However, real-world patients are characterized by dismal characteristics compared to patients in clinical trials. In particular, data on relevant subgroups such as the response for patients with intermediate or poor IMDC or the presence of bone metastasis are still missing. In this multicenter study, we evaluated the safety and effectiveness of cabozantinib plus nivolumab in real-world cohorts from centers in Germany, Switzerland, and Austria.

## 2. Materials and Methods

### 2.1. Patient Cohort and Treatment

Clinical data were collected from twelve oncological centers (in alphabetical order: Cologne, Düsseldorf, Essen, Hannover, Heidelberg, Herne, Jena, Munich, Münster, Regensburg, St. Gallen, and Vienna). Data were retrospectively retrieved from medical records. Patients with advanced or mRCC were eligible. Patients receiving cabozantinib and nivolumab as a standard first-line treatment were included. Treatment was applied according to local standards. Cabozantinib 40 mg orally plus nivolumab 240 or 480 mg i.v. was mandatory and administered according to routine care. Dose adjustments were made at the discretion of the investigators. In total, 21.9% (*n* = 21) required dose reductions or interruptions.

Throughout the duration of this study, the ethical standards of the institutional and/or national research committee and the 1964 Declaration of Helsinki, as subsequently amended, were adhered to. This study was approved by the local Ethics Committee of the Medical Faculty of the University of Duisburg-Essen (22-10567-BO).

### 2.2. Statistical Analysis

Pseudonymized data sets were analyzed using SPSS-Statistics 29.0 (Armonk, NY, USA). The primary outcome measures were the best overall objective response rate (ORR) and PFS. PFS was defined as the time from the start of therapy to the date of radiologic or clinical progression or death. ORR was evaluated by investigators according to the Response Evaluation Criteria in Solid Tumors version 1.1 (RECIST 1.1). Adverse events (AEs) were defined according to the Common Terminology Criteria for Adverse Events (CTCAE) version 5.0. For survival analyses, the Kaplan–Meier method was used. The duration of the follow-up time was calculated from the date of treatment initiation to either the date of death or censored at the last-known follow-up. Overall, two-sided *p*-values ≤ 0.05 were considered statistically significant.

## 3. Results

### 3.1. Patient Characteristics

A total of 96 patients who received the combination of cabozantinib plus nivolumab were identified. The baseline characteristics are displayed in Table 1. The median age at the initiation of therapy was 66.0 years (range 22–85 years). In total, 11.5% of patients were ≥75 years at the start of treatment with cabozantinib plus nivolumab; 66.6% of patients were male. The ECOG performance status was 0–1, 2–4, and unknown in 68.8% (*n* = 66), 14.6% (*n* = 14), and 16.7% (*n* = 16) of patients, respectively. The IMDC risk was favorable, intermediate, poor, or unknown in 22.9% (*n* = 22), 42.7% (*n* = 41), 16.7% (*n* = 16), and 17.7% (*n* = 17) of patients.

The most prevalent histology type was clear-cell RCC (ccRCC) in 63.4% of patients. In total, 60.4% of patients had received prior local treatment. The sites of metastases were lung (55.2%), lymph nodes (50%), bone (40.6%), liver (19.8%), adrenal (14.6%), and brain (13.5%).

### 3.2. Efficacy

The median follow-up from the start of cabozantinib plus nivolumab was 12.7 months (95% CI, 10.0–15.3). ORR was 45.8% (44/96), and the disease control rate (DCR) was 78.1% (75/96, Table 2). The best response to treatment was CR in two patients.

**Figure 1:**
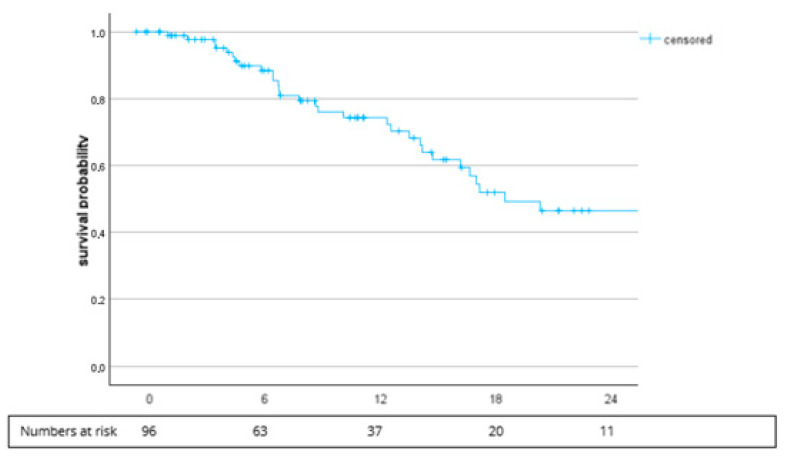
Kaplan–Meier plot for the PFS of all patients. The median PFS was 18.6 months (95% CI, 10.0–27.1).

The median PFS (mPFS) was 18.6 months (95% CI, 10.0–27.1), and the probability of PFS at 6 months was 89.8% (74.3% at 12 months) (Figure 1). The probability of OS at 12 months was 77.5% with cabozantinib plus nivolumab. The median OS was not reached.

**Figure 2:**
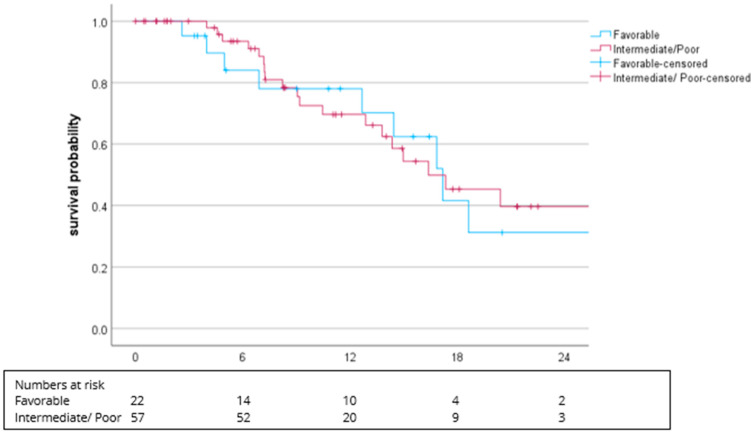
Kaplan–Meier plot for PFS in regard to favorable vs. intermediate/poor IMDC. The median PFS was 17.2 months (95% CI: 13.4–21.0) vs. 16.3 months (95% CI: 10.6–22.1), *p* = 0.742.

The median PFS in patients with favorable IMDC was 17.2 months (95% CI: 13.4–21.0) compared to 16.3 months (95% CI: 10.6–22.1, *p* = 0.742) for intermediate/poor IMDC (Figure 2). 

**Figure 3:**
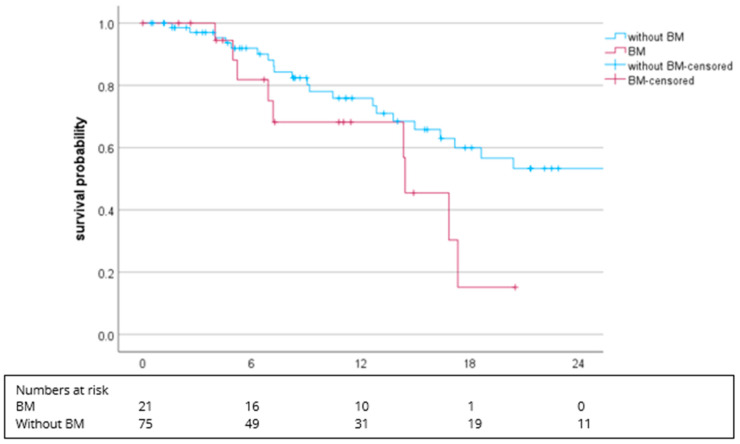
Kaplan–Meier plot for PFS in regard to bone metastasis (BM) vs. without BM. The median PFS was 25.8 months (95% CI: 11.6–40.0) vs. 14.4 months (95% CI: 11.6–17.3), *p* = 0.064.

In patients with bone metastases, the mPFS was 14.4 months (95% CI: 11.6–17.3) and 25.8 months (95% CI: 11.6–40.0; *p* = 0.064) in patients without bone metastasis (Figure 3).

**Figure 4:**
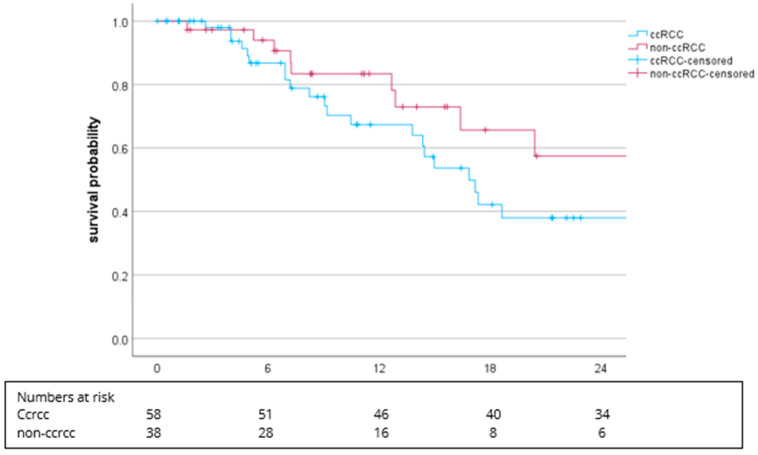
Kaplan–Meier plot for PFS in regard to ccRCC vs. non-ccRCC. The median PFS was 16.8 months (95% CI: 13.5–20.2) vs. 30.6 months (95% CI: 9.1–52,0), *p* = 0.133.

Considering the histology, the mPFS was 16.8 months (95% CI: 13.5–20.2) in ccRCC patients (including ccRCC with sarcomatoid differentiation) and 30.6 months (95% CI: 9.1–52.0, *p* = 0.133) for patients with non-ccRCC subtypes (Figure 4).

### 3.3. Safety/Adverse Events

In total, 82.3% and 41.7% of patients experienced all grades and grade 3–5 AEs, respectively (Table 3).

AEs of any cause led to discontinuation in 25% of patients. Overall, one death was considered to be treatment related (pneumonia) by investigators.

The most observed treatment-related AEs were increased liver enzymes in 34.4% (all grades) and 13.5% (grades 3–4) of patients, respectively, followed by diarrhea with 31.3% for all grades and 8.3% for grades 3–4. Skin toxicity including palmar–plantar erythrodysesthesia (PPE) was noted in 29.2% of patients (all grades) (Table 4).

## 4. Discussion

To our knowledge, this is the first retrospective multicenter study exploring the outcomes of real-world patients treated with the ICI combination of cabozantinib plus nivolumab in a first-line therapy setting.

We report the data of 96 patients from 12 academic hospitals in Germany, Switzerland, and Austria.

With a median age of 66.0 years at the start of treatment, our cohort is older than those in the CheckMate 9ER trial. Furthermore, our cohort differs from the CheckMate 9ER trial in different aspects. Firstly, we have included patients with non-ccRCC. Secondly, we identified only 40 patients (41.7%) with ECOG 0. Thus, a larger proportion of this cohort had a worse performance status compared to 80% of patients with a Karnofsky performance status of 90–100% (corresponds to ECOG 0) in the CheckMate 9ER trial at therapy initiation. However, the median PFS in the overall cohort was 18.6 months, which was higher than reported in 55-month follow-up of the CheckMate 9ER trial [23]. However, our results are consistent with previous data that suggests cabozantinib may enhance immune-checkpoint inhibition [10,11,12,13,25].

Interestingly, the mPFS was similar between patients with a favorable IMDC risk (mPFS 17.2 months) and intermediate/poor IMDC risk (mPFS 16.3 months). The current update of the CM9 trial shows significant differences between these two groups in favor of favorable IMDC [26]. The proportion of patients with an unknown IMDC status (mPFS 30.6 months) may have influenced our result. In our cohort, the mPFS was significantly different between the subgroups with ccRCC (16.8 months) and non-ccRCC (30.6 months). The ccRCC subgroup included ccRCC with sarcomatoid differentiation (*n* = 8; 8.3%), which is known to exert explicit sensitivity for ICI therapies. However, the small number of patients with sarcomatoid differentiation (mPFS 6.9 months) may have had an impact on our results. Our RW results were comparable to the findings of a phase II trial, which tested cabozantinib plus nivolumab in patients with non-ccRCC subtypes [24].

The presence of bone metastasis in RCC is a well-known risk factor according to a lower life expectancy and loss of quality of life [27,28,29]. In this analysis, we have demonstrated that patients with bone metastases had a shorter PFS than patients without metastases (14.4 vs. 25.8 months).

The reported toxicity was lower in our cohort when compared to the prospective CheckMate 9ER trial (82.3% for all grades and 41.7% for grade 3–5 treatment-related adverse events versus 99.7% and 75.3%). This can be explained by the retrospective character of the study and the focus on clinically relevant AEs in routine practice. All 12 sites are experienced clinical trial units which regularly participate in clinical trials and are familiar with CTCAE reporting. However, in daily clinical practice, the documentation of AEs seems to be limited to those that most severely affect patient quality of life.

While some AEs, such as hypertension, were noted in at least 34.7% of patients in the prospective CheckMate 9ERtrial (resulting in it being the third most common adverse event), only 11 patients (11.5%) had documented hypertension in our cohort. In contrast, elevated liver enzymes were reported in a higher fraction of patients compared to the aforementioned prospective trial [34.4% vs. 28.1% (ALT level)/25.3% (AST level)]. Due to differences in reporting, these numbers are difficult to compare.

Our RW data also showed that patients should be informed about diarrhea as one of the most common side effects, which is comparable to the CheckMate 9ER trial (diarrhea grade 1–2 in 53% of patients and grade 3 in 6% of patients). PPE was a relevant finding and affected 29.2% of our patients, which was grade 3–4 in nine of our patients. In addition, we observed one fatal case of pneumonia.

The limitations of our study are its retrospective character with selection and recall bias as well as the short follow-up time. We did not utilize a radiologic blinded independent central review in our analysis. Moreover, the study population and subgroups are too small to perform an informative multivariable analysis.

## 5. Conclusions

In summary, our real-word data support the promising efficacy data of the pivotal trials, particularly in patients with non-ccRCC and those without bone metastases at the start of treatment. The toxicities of cabozantinib plus nivolumab were manageable. However, special attention should be paid to liver, skin, and gastrointestinal toxicities.

## Figures and Tables

**Table 1:** Characteristics of patients. Abbreviations: no—number; ccRCC—clear-cell renal cell carcinoma.

Characteristic, No. (%)	All Patients (*N* = 96)	Patients with ccRCC (*N* = 61)	Patients with Non-ccRCC (*N* = 35)
Median age at start of nivolumab/cabozantinib in years (range)	66.0 (22–85)	67.1 (35–85)	65.4 (22–78)
Age < 75 years	80 (83.3)	53 (86.9)	27 (77.1)
Age ≥ 75 years	11 (11.5)	5 (8.2)	6 (17.1)
Unknown	5 (5.2)	3 (4.9)	2 (5.7)
Gender			
Male	64 (66.6)	39 (63.9)	25 (71.4)
Female	32 (33.3)	22 (36.1)	10 (28.6)
Prior definitive treatment			
Yes	58 (60.4)	37 (60.6)	21 (60)
No	38 (39.6)	24 (39.4)	14 (40)
T-status			
T1	4 (4.2)	0 (0)	4 (11.4)
T2	8 (8.3)	6 (9.8)	2 (5.7)
T3	24 (25)	18 (29.5)	6 (17.1)
T4	12 (12.5)	7 (11.5)	5 (8.2)
Unknown	48 (49.0)	30 (49.2)	18 (51.4)
N-status			
N0	31 (32.2)	21 (34.4)	10 (28.6)
N1 (regional)	19 (19.8)	13 (21.3)	6 (17.1)
N2 (non-regional)	29 (30.2)	19 (31.1)	10 (28.6)
Unknown	17 (17.7)	8 (13.1)	9 (25.7)
M-Status			
M0	12 (12.5)	8 (13.1)	4 (11.4)
M1	84 (87.5)	53 (86.9)	31 (88.5)
ECOG performance status score			
0	40 (41.7)	24 (39.4)	16 (45.7)
1	26 (27.1)	11 (18.0)	15 (42.9)
2	8 (8.3)	8 (13.1)	0 (0)
3	6 (6.3)	6 (9.8)	0 (0)
4	0 (0)	0 (0)	0 (0)
Unknown	16 (16.7)	12 (19.7)	4 (6.5)
IMDC			
Favorable (0 points)	22 (22.9)	18 (29.5)	4 (11.4)
Intermediate (1–2 points)	41 (42.7)	23 (37.7)	18 (51.4)
Poor (≥3 points)	16 (16.7)	13 (21.3)	3 (8.6)
Unknown	17 (17.7)	7 (11.4)	10 (28.6)
Site of metastases			
Lymph nodes	48 (50)	32 (52.4)	16 (45.7)
Lung	53 (55.2)	40 (65.6)	13 (37.1)
Bone	39 (40.6)	25 (41.0)	14 (40)
Liver	19 (19.8)	8 (13.1)	11(31.4)
Adrenal	14 (14.6)	12 (19.7)	2 (5.7)
Brain	13 (13.5)	8 (13.1)	5 (14.2)
Other	15 (15.6)	12 (19.7)	3 (8.6)

**Table 2:** Efficacy of treatment with cabozantinib + nivolumab. Abbreviations: CR—complete remission; DCR—disease control rate; ORR—overall response rate; PD—progressive disease; PR—partial remission; SD—stable disease.

No. (%)	All Patients (*N* = 96)
ORR	44 (45.8)
CR	2 (2.0)
PR	42 (43.8)
SD	31 (32.3)
PD	8 (8.3)
Not evaluable	13 (13.5)
DCR	75 (78.1)

**Table 3:** Summary of treatment-related adverse events (TRAEs).

No. (%)	All Patients (*N* = 96)
Treatment-related adverse events, any grade	79 (82.3)
Grade ≥ 3 TRAEs	40 (41.7)
Treatment-related adverse events resulting in any treatment discontinuation	24 (25)
Treatment-related adverse events leading to death	1 (1.0)

**Table 4:** Summary of treatment-related adverse events.

No. (%)	All Grades	Grades 3–5
Increased liver enzymes	33 (34.4)	13 (13.5)
Diarrhea	30 (31.3)	8 (8.3)
Palmar–plantar erythrodysesthesia	28 (29.2)	9 (9.4)
Fatigue	18 (18.8)	3 (3.1)
Hypothyroidism	12 (12.5)	8 (8.3)
Nausea	11 (11.5)	1 (1.0)
Hypertension	11 (11.5)	8 (8.3)
Mucosal inflammation	10 (10.4)	3 (3,1)
Loss of weight	8 (8.3)	2 (2.1)
Thrombopenia	7 (7.3)	1 (1.0)
Leukopenia	5 (5.2)	1 (1.0)
Anemia	5 (5.2)	1 (1.0)

## Data Availability

The datasets generated for this study are available on request to the corresponding author.
